# Quantitative Proteomic Analyses of a Pathogenic Strain and Its Highly Passaged Attenuated Strain of* Mycoplasma hyopneumoniae*

**DOI:** 10.1155/2019/4165735

**Published:** 2019-07-01

**Authors:** Sha Li, Liurong Fang, Wei Liu, Tao Song, Fuwei Zhao, Ruoxi Zhang, Dang Wang, Shaobo Xiao

**Affiliations:** ^1^State Key Laboratory of Agricultural Microbiology, College of Veterinary Medicine, Huazhong Agricultural University, Wuhan 430070, China; ^2^The Key Laboratory of Preventive Veterinary Medicine in Hubei Province, The Cooperative Innovation Center for Sustainable Pig Production, Wuhan 430070, China; ^3^Key Laboratory of Prevention and Control Agents for Animal Bacteriosis (Ministry of Agriculture), Institute of Animal Husbandry and Veterinary Sciences, Hubei Academy of Agricultural Sciences, Wuhan 430070, China

## Abstract

*Mycoplasma hyopneumoniae* is the causative agent of porcine enzootic pneumonia, a chronic respiratory disease in swine resulting in enormous economic losses. To identify the components that contribute to virulence and unveil those biological processes potentially related to attenuation, we used isobaric tags for relative and absolute quantification technology (iTRAQ) to compare the protein profiles of the virulent* M. hyopneumoniae* strain 168 and its attenuated highly passaged strain 168L. We identified 489 proteins in total, 70 of which showing significant differences in level of expression between the two strains. Remarkably, proteins participating in inositol phosphate metabolism were significantly downregulated in the virulent strain, while some proteins involved in nucleoside metabolism were upregulated. We also mined a series of novel promising virulence-associated factors in our study compared with those in previous reports, such as some moonlighting adhesins, transporters, lipoate-protein ligase, and ribonuclease and several hypothetical proteins with conserved functional domains, deserving further research. Our survey constitutes an iTRAQ-based comparative proteomic analysis of a virulent* M. hyopneumoniae* strain and its attenuated strain originating from a single parent with a well-characterized genetic background and lays the groundwork for future work to mine for potential virulence factors and identify candidate vaccine proteins.

## 1. Introduction

*M. hyopneumoniae* is the causative agent of porcine enzootic pneumonia, which is a worldwide epidemic that can cause enormous economic losses as a result of retarded growth in pigs and the cost of disease control [1]. Despite its low direct mortality,* M. hyopneumoniae* increases the host's susceptibility to secondary respiratory infections by damaging cilia and epithelial cell, resulting in aggravated lung lesions and fatal respiratory diseases [2]. To date, the determination of the potential molecular mechanisms of the pathogenicity of* M. hyopneumoniae* has been hampered by the fastidious growth requirements of this organism and the lack of tools for its genetic manipulation.

Comparative genomic analyses have been performed to reveal the genetic basis of virulence attenuation of* M. hyopneumoniae* and to predict potential virulence factors [3–5]. However, in the case of* M. hyopneumoniae*, the different strains share highly similar genome structures and gene orders, and few differences have been detected among them at the genomic level. Thus, we decided to turn our attention to the variations in expression levels of the predicted virulence factors.

Previous studies have described the transcriptome changes under different growth conditions [6–9] and with changes in gene regulation among different mycoplasma species [10]. No transcriptomic studies comparing virulent and avirulent* M. hyopneumoniae* strains have been published. The comparative proteomic reports of pathogenic and nonpathogenic strains based on two-dimensional gel electrophoresis have revealed a few differentially expressed proteins [11–13]. However, gel-based proteomic methods are usually hindered by their low-throughput and the difficulty of direct quantitative comparison between samples. Recently, Paes et al. adopted cell fractioning technology coupled with mass spectrometry, providing much more potential virulence factor/vaccine candidates than conventional ones through comparative whole cell proteome profiles analysis of two* M. hyopneumoniae* strains and* M. flocculare* [14].

In this study, we undertook a large-scale proteomic comparison between the virulent* M. hyopneumoniae* strain 168 and its highly passaged attenuated vaccine strain 168L and identified differentially displayed proteins using the nongel-based isobaric tags for relative and absolute quantification (iTRAQ) approach. This survey supplied a comprehensive iTRAQ-based comparative proteomic analysis of a pathogenic and a nonpathogenic* M. hyopneumoniae* strain, both originating from one parent strain with a well-characterized genetic background. This approach eliminated the effects caused by differences at the genome level, and it demonstrated some novel promising virulence-related proteins in these strains compared with previous researches.

## 2. Materials and Methods

### 2.1. Ethics Statement

The animal experiments in this study were approved by The Scientific Ethic Committee of Huazhong Agricultural University, Wuhan, China (Approval Number: HZAURAB-2017-008) and conducted in accordance with the Hubei Regulations for the Administration of Affairs Concerning Experimental Animals.

### 2.2. Mycoplasma Strains and Their Cultivation

*M. hyopneumoniae* strains 168 and 168L were acquired from the Jiangsu Academy of Agricultural Sciences (Nanjing, China).* M. hyopneumoniae* strain 168 is a pathogenic strain isolated in China in 1974, leading typical mycoplasmal pneumonia of swine [5, 15]. The stable attenuated strain 168L was obtained after 380 continuous serial passages in KM2 cell-free medium (a modified Friis medium) and has been developed into a commercially available vaccine against* M. hyopneumoniae* in China [5, 16]. Cultures were maintained in KM2 cell-free medium at 37°C. For the proteomic study, 1.5 L cultures of strains 168 and 168L were grown to late-log phase as described by Calus [17]. Three replicate samples of each culture were harvested by centrifugation at 1,2000×g for 30 minutes at 4°C and washed three times with ice-cold PBS.

### 2.3. Protein Extraction, Digestion, and Labeling with iTRAQ Reagents

Bacterial cells were resuspended in lysis buffer (7 M urea, 2 M thiourea, 4% CHAPS, 40 mM Tris-HCl, pH 8.5) containing 1 mM PMSF, 2 mM EDTA, and 10 mM DTT. The samples were then sonicated for further protein solubilization. After centrifugation (25,000×g, 4°C, 20 min), the protein concentration of the supernatants was determined using the Bradford assay. Each sample (100 *μ*g) was digested with trypsin (Promega, Madison, WI, USA) at 37°C for 12 h. The three lysate replicates of* M. hyopneumoniae* strain 168 were labeled with iTRAQ reagents 113, 114, and 115, and of strain 168L with iTRAQ reagents 116, 117, and 119.

### 2.4. SCX Chromatography

iTRAQ-labeled mixed peptides were fractionated by SCX chromatography using a 20AB HPLC system (Shimadzu, Kyoto, Japan). Peptide mixture was reconstituted in buffer A (25 mM NaH_2_PO_4_ in 25% acetonitrile, pH2.7) and loaded onto an UltremexSCX column (4.6 × 250 mm). The loaded peptides were eluted at a flow rate of 1 ml/min with 5% buffer B (25 mM NaH_2_PO_4_, 1 M KCl in 25% acetonitrile, pH 2.7) for 7 min, a linear gradient of 5–60% and 60-100% buffer B for 20 min and 2 min, respectively, and 100% buffer B for 1 min and then 5% buffer B for 10 min. The chromatograms were recorded at 214 nm. In total, 20 fractions were collected, desalted with a StrataX desalting column, and dried using vacuum centrifugation.

### 2.5. LC-MS/MS Analysis

The lyophilized fractions were resuspended in buffer A (5% acetonitrile, 0.1% formic acid) and loaded on a Symmetry C18 trapping column (5 *μ*m particles, 180 *μ*m inner diameter × 20 mm; Waters) for online trapping and desalting. Then the peptides were eluted from the trapping column over a BEH 130 C18 column (1.7 *μ*m particles, 100 *μ*m inner diameter × 100 mm) with a gradient buffer B (95% acetonitrile, 0.1% formic acid) at 300 nL/min (5-35% B, 35 min; 35-60% B, 5 min; 80% B, 2 min; 80-5% B, 1 min; 5% B, 10 min).

MS/MS was operated with a Triple TOF 5600 mass spectrometer (AB Sciex, Concord, ON). Data were acquired using a nanospray voltage of 2.5 kV, an interface heater temperature of 150°C, curtain gas at 30 psi, and nebulizer gas at 15 psi. Survey scans were acquired in 250 ms and as many as 30 product ion scans were collected if exceeding the threshold of 120 counts per second (cps) with +2 to +5 charge states. Total cycle time was fixed at 3.3 s. Dynamic exclusion was set for 1/2 of peak width (18 s) and the fragmentation energy was set to 35 ± 5 eV.

### 2.6. Protein Identification and Quantification

Tandem mass spectra were extracted by AB SCIEX MS Data Converter version 1.3. All MS/MS data were analyzed using the Mascot search algorithm (Matrix Science, London, UK; version b2.5.1) against the NCBI* M. hyopneumoniae* 168 & 168L database (201607, 3408 entries) with trypsin as the digestion enzyme. For protein identification, Mascot was searched with a parent ion tolerance of 15 ppm and a fragment ion mass tolerance of 0.050 Da. Carbamidomethyl of cysteine and iTRAQ8plex of lysine and the n-terminus were specified in Mascot as fixed modifications, while oxidation of methionine and iTRAQ8plex of tyrosine were allowed as variable modifications.

MS/MS-based peptide and protein identifications were validated using Scaffold (version Scaffold_4.6.1, Proteome Software Inc., Portland, OR). Peptide identifications were accepted if they could be established with less than 1% false discovery rate (FDR) by the Scaffold Local FDR algorithm, and each identified protein involved at least one unique peptide.

Scaffold Q+ (version Scaffold_4.6.1, Proteome Software Inc., Portland, OR) was used to quantitate the isobaric tag peptide and protein identifications. Normalization was performed iteratively (across samples and spectra) on intensities. Medians were used for averaging. Spectra data were log-transformed, pruned of those matched to multiple proteins, and weighted by an adaptive intensity weighting algorithm. A protein was considered to be differentially displayed if it contained at least two unique peptides with a minimum fold change of 0.585 on the log⁡2 scale, and Mann–Whitney Test with an unadjusted significance level p<0.05 corrected by the Benjamini-Hochberg method.

### 2.7. Bioinformatic Analysis of Proteins

Gene Ontology (GO) analysis was performed to map all of the identified proteins to GO terms in database (http://www.geneontology.org/). The metabolic pathway of identified proteins was performed using the Kyoto Encyclopedia of Genes and Genomes (KEGG) database (https://www.genome.jp/kegg/). The significantly enriched GO terms or pathways of differentially expressed proteins in the background of the identified proteins were determined by a hypergeometric test (p<0.05).

The physical and chemical parameters of the given proteins were computed via ProtParam tool (https://web.expasy.org/protparam/) [18]. Lipoprotein signal peptides were predicted by LIPO (http://services.cbu.uib.no/tools/lipo) [19] and LipoP 1.0 Server (http://www.cbs.dtu.dk/services/LipoP/) [20]. Subcellular location of proteins was predicted by using PSORTb version 3.0.2 (https://www.psort.org/psortb/index.html) [21] and CELLO v.2.5 (http://cello.life.nctu.edu.tw/) [22]. TMHMM v2.0 (http://www.cbs.dtu.dk/services/TMHMM-2.0/) and SignalP v.4.1 (http://www.cbs.dtu.dk/services/SignalP/) were used for predicting transmembrane helices and the presence and location of signal cleavage sites, respectively [23, 24]. SecretomeP 2.0 server (http://www.cbs.dtu.dk/services/SecretomeP/) was used to identify nonclassical protein secretion, i.e., not signal peptide triggered protein secretion [25]. Uncharacterized/hypothetical proteins were subjected to BlastP (https://www.ncbi.nlm.nih.gov/), Pfam 31.0 web servers (http://pfam.xfam.org/) [26] and InterPro (http://www.ebi.ac.uk/interpro/) [27] to examine conserved functional domains for putative function assignment. For identifying proteins associated with virulence we used VirulentPred web server (http://203.92.44.117/virulent/index.html) [28].

### 2.8. Gene Cloning, Expression, and Purification of Recombinant Proteins

Full-length genes of the four selected proteins were amplified directly from* M. hyopneumoniae* strain 168 genomic DNA using primer pairs in Table 1. Mycoplasmas use UGA as tryptophan codon while* E. coli* retain it as a stop codon. In order to mutate TGA to TGG and achieve mycoplasma proteins' heterologous expression, we used specific primers (Table 1) to conduct overlapping PCR for site-directed mutagenesis. Then the products were cloned into pET-28a vector and transformed into* E. coli* DH5*α*. After checking the inserts by sequencing, the reconstructed plasmids were transformed into* E. coli* BL21 (DE3) to express the N-terminal 6×His-tagged recombinant proteins. Obtained proteins were purified by nickel affinity chromatograph (GE Healthcare, Piscataway, NJ, USA), dialyzed in PBS, and concentrated with Amicon Ultra centrifugal filter units (Millipore, Darmstadt, Germany). Protein concentration was determined by BCA Protein Assay Kit (Beyotime, Nanjing, China), and the purity was verified on 12% Coomassie-stained SDS-PAGE gels.

### 2.9. Production of Polyclonal Antibodies

Polyclonal antisera were produced in New Zealand White rabbits via four times subcutaneous immunization at 2-week intervals. Rabbits were first immunized with 1mg of purified proteins mixed with equal volumes of complete Freund's adjuvant (Sigma, St. Louis, MI, USA). The rest three booster immunizations used 1mg of purified proteins suspended in equal volumes of incomplete Freund's adjuvant (Sigma, St. Louis, MI, USA). Ten days after the last immunization, blood samples were collected via cardiac bleeding and centrifuged to obtain serum.

### 2.10. Western Blot Analysis

Western workflow utilizing stain-free technology was performed according to the supplier protocol. Samples of 10 ug were separated in 12% TGX Stain-Free Fastcast acrylamide gels (Bio-Rad, Hercules, CA, USA). After the electrophoresis, gels were placed in the ChemiDoc™ Touch Imaging System (Bio-Rad, Hercules, CA, USA) and activated for 5 min with UV treatment to visualize total proteins. Then the proteins were transferred to PVDF membranes. After blocking with 10% milk for 3 h at room temperature, membranes were incubated at room temperature for 4 h with rabbit polyclonal antibodies (1:500) against methylmalonate-semialdehyde dehydrogenase, endo-1,4-beta-glucanase, enolase, and translation elongation factor Tu. Goat anti-rabbit IgGs conjugated with horseradish peroxidase (1:4000; Beyotime, Nanjing, China) were used as secondary antibodies, and, after antibodies incubation, stain-free blot images were captured for total protein loading control and normalization. Then proteins were detected with ClarityTM ECL reagents (Bio-Rad, Hercules, CA, USA), and the intensity values of the target bands were normalized to that of the total proteins in the lane. The 168/168L intensity ratios were calculated for each protein, and three replicates were subjected to statistical analysis.

## 3. Results

### 3.1. Global Protein Profiles Overview

To establish more detailed comparable proteome profiles of the virulent strain 168 and its attenuated strain 168L, whole proteins were extracted from the two strains, digested and labeled with iTRAQ reagents, followed by downstream analysis LC-MS/MS.

**Figure 1:**
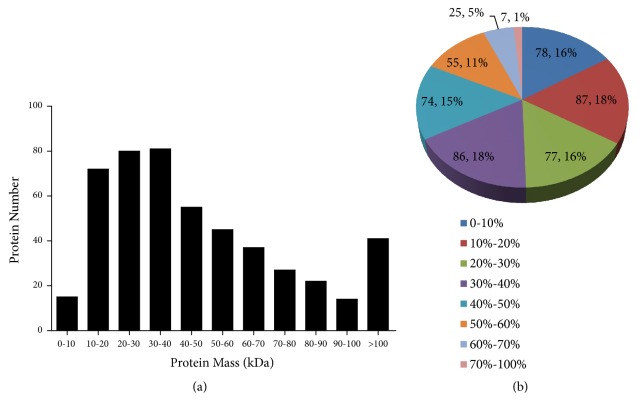
Overview of all proteins identified in* M. hyopneumoniae*. (a) Distribution of proteins of different molecular weights. (b) Coverage of proteins by LC-MS/MS identified peptides.

Results of the iTRAQ assay showed that 489 proteins, 70% (489/695) of the coding sequences in* M. hyopneumoniae*, were identified from 30,214 spectra from the whole output of 322,266 spectra with an FDR of 1% (Table S1) [15]. The molecular weights of most of the identified proteins ranged between 10 and 60 kDa (68%, Figure 1(a)). 50.5% of the identified proteins possessed sequence coverage of the identified peptides of higher than 30% (Figure 1(b)).

**Figure 2:**
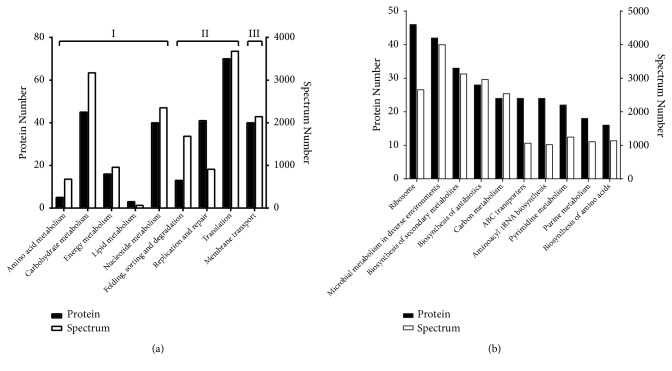
KEGG classification of the annotated proteins and corresponding spectra in quantitative proteome of* Mycoplasma hyopneumoniae *168 and 168L. (a) KEGG classification of the annotated proteins and corresponding spectra based on secondary pathway hierarchy: (I) metabolism; (II) genetic information and processing; (III) environmental information and processing. (b) The 10 most highly represented KEGG pathways of the annotated proteins.

Of the 489 proteins identified, 273 proteins were annotated into 9 groups based on the KEGG categories. The categories with the highest abundance were translation (70 proteins), carbohydrate metabolism (45 proteins), replication and repair (41 proteins), nucleotide metabolism (40 proteins), and membrane transport (40 proteins) (Figure 2(a)). Meanwhile, further classification indicated that the top ten pathways in which the identified proteins were involved were ribosome biosynthesis, microbial metabolism in diverse environments, biosynthesis of secondary metabolites, biosynthesis of antibiotics, carbon metabolism, ABC transporters, aminoacyl-tRNA biosynthesis, pyrimidine metabolism, purine metabolism, and biosynthesis of amino acids (Figure 2(b)).

### 3.2. Differentially Expressed Proteins

We detected and quantified 489 proteins, of which 35 were more abundant in the Mhp168 strain and 35 in the Mhp168L strain, based on having a log⁡2 ratio ≥ 0.585 or ≤-0.585 and a statistically significant differences at p<0.05 (Table 2).

**Figure 3:**
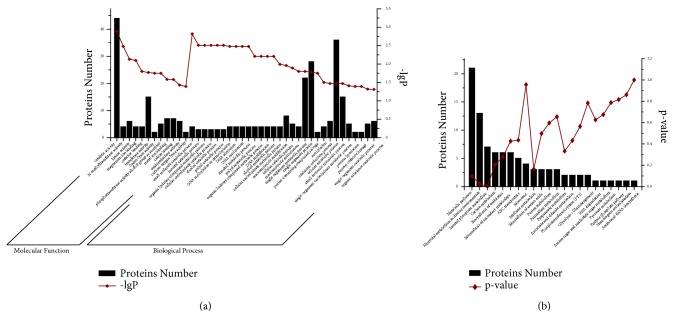
Functional categories of differentially expressed proteins. (a) GO term enrichment analysis of the annotated differentially expressed proteins for the categories “molecular function” and “biological process”. (b) Pathway enrichment analysis of the annotated differentially expressed proteins.

Gene Ontology (GO) enrichment analysis was then performed for the differentially expressed proteins for the categories “molecular function” (F), “cellular components” (C), and “biological process” (P) (Table S2).  Figure 3(a) shows the result of the enrichment analysis for these functions (GO terms). A total of 12 GO terms were enriched for molecular function and 30 for biological processes, while there's no statistically significant results for cellular components. For the GO category “molecular function”, significant synthesis of the proteins related to catalytic activity were detected. In addition, the GO terms “N-methyltransferase activity”, “kinase activity”, and “magnesium ion binding” were also highly enriched. The highest enrichment for biological processes was associated with small molecule catabolism. In addition, the GO terms “inositol catabolic process”, “organic hydroxy compound catabolic process”, “cellular carbohydrate catabolic process”, “alcohol catabolic process”, “polyol catabolic process”, “DNA methylation or demethylation”, “DNA alkylation”, “DNA methylation”, and “inositol metabolic process” were also highly enriched.

To examine which biological pathways were apparently altered, we carried out a pathway analysis. Enriched pathways were grouped into 2 categories with P value <0.05 (Table S3, Figure 3(b)). The two pathways were involved in microbial metabolism in diverse environments and in inositol phosphate metabolism.

### 3.3. Expression of Recombinant Proteins and Preparation of Polyclonal Antibodies

**Figure 4:**
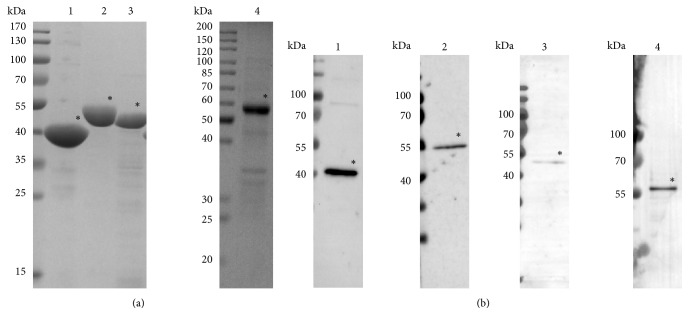
The expression of recombinant proteins and their reactivity with corresponding antisera. (a) Coomassie blue stained SDS-PAGE gel of purified recombinant proteins. (b) Western bolt analyses of purified proteins using their corresponding antisera. Lane 1, endo-1,4-beta-glucanase; lane 2, enolase; lane 3, translation elongation factor Tu; and lane 4, methylmalonate-semialdehyde dehydrogenase.

Four differently expressed proteins, endo-1,4-beta-glucanase, enolase, translation elongation factor Tu, and methylmalonate-semialdehyde dehydrogenase were chosen to verify the proteomic differences. Within the virulent strain 168 samples, endo-1,4-beta-glucanase, enolase, and translation elongation factor Tu were more abundant, which might conduce to the virulence of* M. hyopneumoniae*. All these three proteins are cytosolic enzymes, lacking transmembrane domains or traditional signal sequences (Table 3), yet intensive researches during the past years have suggested that they could be detected on microbe surface and moonlight as adhesins [29–34]. On one hand we expect to obtain sera to validate the comparative results and on the other hand we are calculated to conduct further experiments around these proteins. We expressed these recombinant proteins in prokaryotic system (Figure 4(a)) then got the rabbit sera against the proteins, respectively (Figure 4(b)).

### 3.4. Validation of Selected Proteins by Western Blot

**Figure 5:**
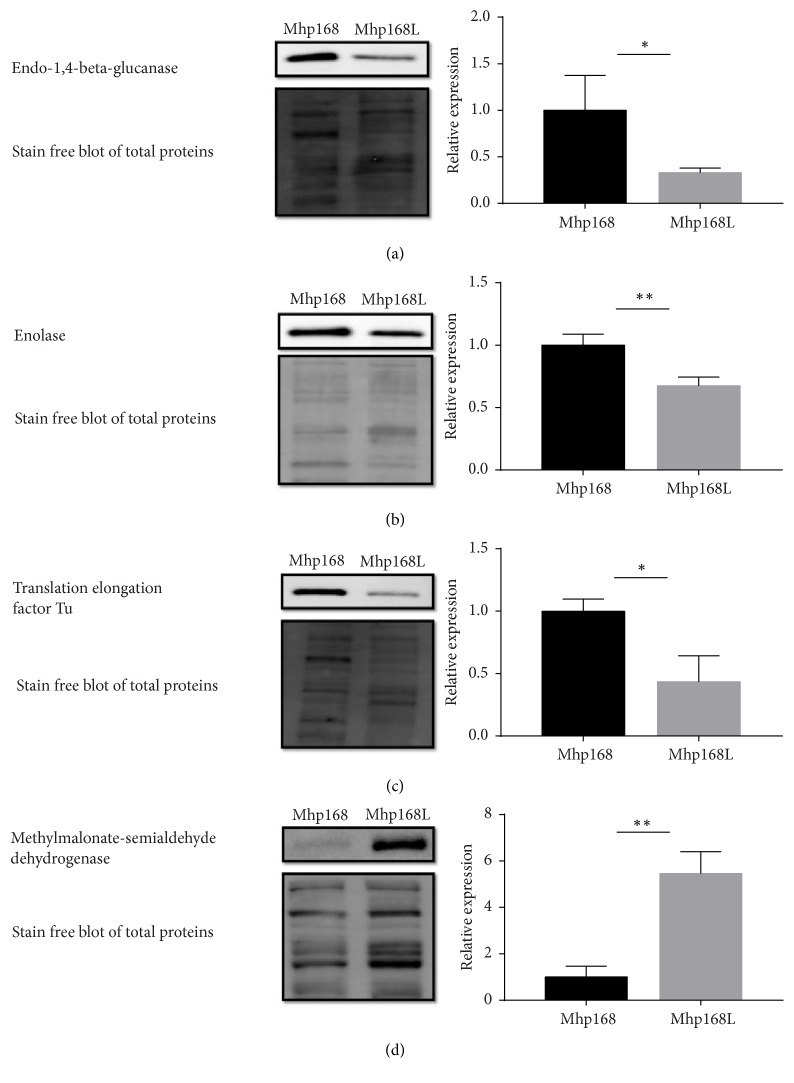
Western blot analyses of differentially expressed proteins using stain-free technology. (a) Endo-1,4-beta-glucanase, (b) enolase, (c) translation elongation factor Tu, and (d) methylmalonate-semialdehyde dehydrogenase. Left: immunoblot images for selected proteins. Total protein loading on the same membrane was used as control. Right: the expression level of the selected protein in strain 168L was presented as the fold change between 168L and 168. Data are present as mean ± SD (n = 3). *∗*p < 0.05, *∗∗* p < 0.01, Student t-test.

We performed western blot analyses using a stain-free technology to assess the levels of four selected differentially expressed proteins. As shown in Figure 5, the alterations in expression levels of these proteins between strain 168 and 168L were consistent with the results from quantitative proteomic analysis, suggesting the credibility of our proteomic data.

## 4. Discussion

In this study, we compared the protein profiles of the pathogenic* M. hyopneumoniae* strain 168 and its highly passaged attenuated strain 168L using iTRAQ strategy for the first time. We identified 70 differentially expressed proteins to mine candidate virulence determinants and proteins or biological processes leading to attenuation.

### 4.1. Proteins Involved in Inositol Phosphate Metabolism

Among all the sequenced mycoplasma species,* M. hyopneumoniae* is the only one having a gene cluster for myo-inositol utilization [35]. A previous report, based on the reconstruction of a genome-scaled metabolic model for three mycoplasmas in silico, suggested that the myo-inositol metabolism may be one of the reasons for the high virulence of* M. hyopneumoniae* compared to that of* M. flocculare* and* M. hyorhinis* [35]. In our study, seven related enzymes of inositol phosphate metabolism, namely, myo-inositol 2-dehydrogenase (MHP168_253), myo-inositol catabolism protein (MHP168_246), myo-inositol 2-dehydrogenase (MHP168_247), methylmalonate-semialdehyde dehydrogenase (MHP168_244), 5-dehydro-2-deoxygluconokinase (IolC), myo-inositol catabolism (IolD), and myo-inositol catabolism protein (IolE), were more abundant in the vaccine strain. These proteins covered all the enzymes comprising the classical myo-inositol bacterial catabolic pathway in* M. hyopneumoniae,* with the exception of the enzyme acting as 5-dehydro-2-deoxyphosphogluconate aldolase (IolJ) whose gene was also absent in its genome. Nevertheless, Ferrarini et al. [36] hypothesized that one gene copy among those annotated as fructose-biphosphate aldolase (Fba) from* M. hyopneumoniae* might function as IolJ from other organisms and confirmed that* M. hyopneumoniae* had been able to utilize myo-inositol from the culture medium.

The increments of those protein levels might facilitate the catabolism of myo-inositol to produce dihydroxyacetone-phosphate (DHAP) and acetyl-coenzyme A (CoA). DHAP can enter glycolysis while acetyl CoA can be widely used in macromolecular biosynthesis and energy production to support cell growth and proliferation. The presence of inositol in mammalian hosts' bloodstream [37] and the degradation of phosphatidylinositol from host' pulmonary surfactant [38] make myo-inositol available for the strains* in vivo*. Considering the fact that myo-inositol has been one stable readily abundant component in the culture medium containing swine serum, we may assume that* M. hyopneumoniae* retains the ability of degrading myo-inositol* in vivo*, then the capability is increased with passage* in vitro*. The vaccine strain might have developed an enhanced ability to cope with differences in environmental carbon sources. However, the myo-inositol utilization gene cluster existed in both strains, and there were no genetic variations in the coding sequences or intergenic regions of those genes [5]. It may be suggested that strains which possess this gene cluster could express it constitutively, while the regulatory mechanism for the enhancements is unclear and still need future research.

### 4.2. Proteins Involved in Nucleotide Metabolism

**Figure 6:**
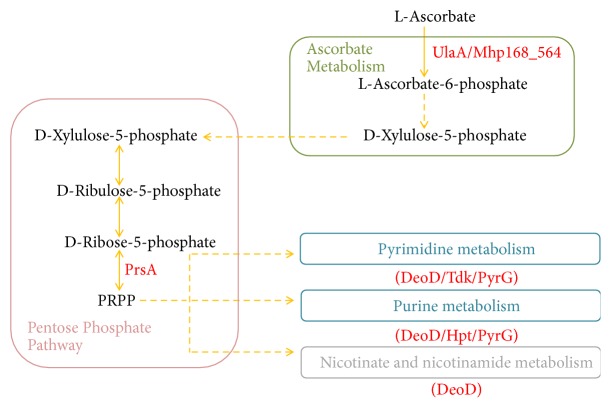
Metabolic pathways for ascorbate, pentose phosphate, nucleotide, and nicotinate related to differentially expressed proteins between* M. hyopneumoniae* strain 168 and 168L. Red text indicates the upregulated proteins in strain 168 compared with that of 168L.

*M. hyopneumoniae* cannot synthesize purines and pyrimidines* de novo*. However, this organism is able to take up exogenous nucleobases and nucleosides, as well as those produced internally by DNA and RNA degradation, and then synthesize nucleotides through salvage pathways and interconversions [39]. In the virulent strain, the upregulation of transport protein SgaT (UlaA) and PTS system galactitol-specific enzyme IIB component (MHP168_564) participated in ascorbate transport suggests the enhanced influx of D-Xylulose-5-phosphate (Xyl5P) through ascorbate metabolism into the pentose phosphate pathway to generate phosphoribosyl pyrophosphate (PRPP) (Figure 6). Meanwhile, the increased expression of ribose-phosphate pyrophosphokinase (PrsA) of the pentose phosphate pathway, catalyzing ribose-5-phosphate (R5P) to PRPP, implies the enriched production of PRPP which is a key intermediate to synthesize purine and pyrimidine nucleotides, as well as nicotinamide adenine dinucleotide (NAD) (Figure 6) [40]. We found that in our data four enzymes participated in nucleoside metabolism: purine-nucleoside phosphorylase (DeoD), hypoxanthine-guanine phosphoribosyl transferase (Hpt), thymidine kinase (Tdk), and CTP synthase (PyrG), all upregulated in the virulent strain. NAD is required for the generation of NADPH that responds to oxidative burst, while synthesis of nucleotides can also promote DNA damage repair caused by oxidative stress [41]. Thus, the increased synthesis of PRPP, NAD, and the upregulation of the nucleotide synthesis in virulent strain 168 could be identified as defense to reactive oxygen species (ROS) damage, fitting well with the idea of an elevated virulence.

### 4.3. Potential Virulence Factors

Mycoplasmas contain a range of virulence factors in their pathogenic machinery, including enzymes, transporters, transcriptional regulators, lipoproteins [42]. Among the overrepresented proteins in virulent strain 168, proteins associated with virulence usually play important roles in* M. hyopneumoniae* offence and defense, meriting more attention. To predict putative novel virulence factors and search for potential drug/vaccine targets, we used VirulentPred to perform the* in silico* analysis for the 35 upexpressed proteins in the virulent strain 168. The server categorized 18 proteins to be associated with virulence. Out of these, 10 proteins are ones with known functions, and the others are termed as “hypothetical” or “uncharacterized”. As a complement to the bioinformatics analyses, we also searched manually the 35 proteins and/or their paralogues for the references that support their virulence-related activities in* M. hyopneumoniae*, or in other pathogens. Then additional 8 proteins classified as “nonvirulent” were recruited into the group of putative virulence proteins. The results are summarized in Table 4 and the listed proteins are discussed in the main text below. 

*Lipoproteins*. Three lipoproteins were identified in this study. MHP168_392 and MHP168_393 were downexpressed and MHP168_418 were upexpressed in the virulent strain 168. Most lipoproteins are surface exposed and important components of mycoplasma membranes, and some in* M. hyopneumoniae* have been identified to play important roles in pathogenesis [43–49]. Mhp378, a homolog of MHP168_392 from* M. hyopneumoniae* 232, has been identified as a species-specific, highly immunogenic membrane-associated protein [50]. The homolog of MHP168_393 in* M. hyopneumoniae* 232 (mhp379) is a surface-exposed exonuclease with probable function in importing nucleic acid precursors [47]. Furthermore, surface located MHP168_418 is shown with the ability to induce apoptosis of porcine peripheral blood mononuclear cells* in vitro* [51].* M. hyopneumoniae* usually cannot employ the similar mechanisms used by other mycoplasma species to generate antigenic diversity through genetic variation [52]. Nonetheless, the posttranslational proteolytic processing, which targeted mycoplasmal membrane lipoproteins and other surface-associated proteins, could create a dynamic surface topograph [53]. The identified lipoproteins with differential expression levels, as well as their probable posttranslational cleavage, could lead to antigenic variations, thereby affecting its immune evasion in host. 

*Adhesion-Related Proteins*. The adherence of* M. hyopneumoniae* to ciliated respiratory epithelium is mainly mediated by the membrane protein P97 [54]. The finding of higher expression levels of P97, protein P97-copy 2, and protein P102-copy 2 in the vaccine strain compared with those of the virulent strain was unexpected, but similar results were also found in the transcriptome comparison between* M. flocculare* and* M. hyopneumoniae* [10]. A previous study indicated that the cilium binding domain of P97 is found exclusively in the R1 region, the functional site requiring a minimum of eight tandem repeating units (AAKPV/E) [54]. However, three transversion mutations (E863V) occurred in the tandem repeating units in P97 of 168L, which might partly affect the adhesion of vaccine strain [5]. P102 accompanies P97 to form a two-gene operon. Both the P97 and P102 genes have several paralogs within the* M. hyopneumoniae* genome [5]. The sequences of the paralogs are uncompleted. Furthermore, the p97-copy 2 protein of both strains lacks the R1 domain while protein P102-copy 2 was also truncated [5]. Thus, a higher abundance of these proteins might not mean a stronger adhesion ability of the vaccine strain. Adherence to ciliated respiratory epithelium is a multifactorial process that also involves other proteins.

In our proteomic data, we detected several upregulated moonlighting proteins in strain 168 that could be used to invade host cells: elongation factor Tu [30, 31], enolase [29, 32, 34], and endo-1,4-beta-glucanase [33]. Intensive reports have described some bacterial metabolic enzymes not only performing key metabolic functions in the cytosol of bacterial cell but also locating on the bacterial surface without a signal sequence, moonlighting as an adhesion contributor to host cells [55]. These multifunctional proteins located on the mycoplasma surface could adhere to the swine tracheal cilia and bind to host factors plasminogen and fibronectin. Mycoplasma surface-bound plasminogen is converted to plasmin by tissue plasminogen activator. Plasmin cleaves host extracellular matrix proteins and activates matrix metalloproteases, assisting the pathogen to invade host issues and providing amino acids for growth of* M. hyopneumoniae* [33]. Fibronectin is widespread in the ciliary borders of the bronchioles and could bind to glycosaminoglycans, collagens, DNA, fibrin, and cell surface integrins, etc. These properties of fibronectin make it a physical bridge between pathogens and host cells [56]. Those mycoplasma surface proteins binding to plasminogen and fibronectin have been suggested to be associated with virulence and warrant further investigation.

Significantly expressed protein O-sialoglycoprotein endopeptidase in strain 168 also regulates the invasion of pathogen via degrading sialoglycosylated host-cell proteins and mediating adhesion to host cells [57, 58]. The traits of these proteins, playing roles in the pathogen-host interactions, affect the pathogenesis of* M. hyopneumoniae* in part. 

*Transporters*. Transporter proteins play important roles in bacteria, transporting various molecules to support survival and growth in different niches [59]. Cation-transporting P-type ATPase (PacL) is a member of transmembrane P-type ATPases, which are involved in transportation of ions and phospholipids, using the energy derived from ATP hydrolysis [60–62]. Mg^2+^ ion transporter (MgtE) is a highly Mg^2+^-selective channel gated by Mg^2+^, transporting substrates across the cytoplasmic membrane by utilizing the electrochemical gradient [63]. Both of MgtE and PacL function in maintaining metal homeostasis in pathogen and survival in host, thus considered as virulence determinants in some bacteria [62–65]. Inactivation of ABC transporters often has deleterious effects on the virulence in bacteria, resulting in attenuated phenotypes and decreased adherence to host cells [59]. ABC transporter ATP-binding-Pr1 (Pr1), ABC transporter protein (MHP168_616), and sugar ABC transporter ATP-binding protein (MHP168_615) may be associated with virulence via participating in the regulation of cation homeostasis or adhesive ability. Pts system lichenan-specific IIa component (LicA) and PTS system galactitol-specific enzyme IIB component (MHP168_564) are predicted to be virulent factors on VirulentPred webserver, and their expression levels in strain 168 were upregulated as compared to 168L, suggesting that these proteins may be suitable targets for antibacterial vaccine and therapies.

*Enzymes*. Two lipoate-protein ligase A (LplA-1 and LplA) were upregulated in the pathogenic strain 168. LplA ligates exogenous lipoic acid to lipoyl domains of certain metabolic enzymes complexes involved in oxidative metabolism [66]. Previous study showed that growth of LplA1-deficient* L. monocytogenes* was damaged specifically in the host and virulence was 1/300th as that of wild-type in animals [67]. Possibly, we speculate that LplA could also act as a virulence factor in* M. hyopneumoniae*.

Enzymes PrsA have been treated as potential targets for therapeutic and vaccine candidates according to previous reports [68]. Cysteinyl-tRNA synthetase is essential for bacteria growth and classified as a “virulent” protein in our predicted results. Increased level of these proteins in virulent strain 168 could support this idea; nonetheless, what kind of part do these proteins play in the pathogenesis and whether they could indeed cause the predicted effects in this strain still needs experimental verification. 

*Transcriptional Regulator*. In strain 168, we found the expression of ribonuclease III (Rnc) was significantly elevated. This ribonuclease is known to be assisting in pathogenesis of other bacteria via regulating the synthesis of virulence factors through RNase III-dependent posttranscriptional manner [69, 70]; its upexpression in virulent strain made us guess that it might also modulate gene expression of virulent factors in* M. hyopneumoniae*, therefore contributing to high virulence. 

*Uncharacterized/Hypothetical Virulence-Related Proteins*. According to the previously published genome data, 268 of 695 (~39%)* M. hyopneumoniae* proteins were not assigned functions and annotated as “uncharacterized” or “hypothetical” proteins [15]. In many sequenced bacterial genomes, the uncharacterized/hypothetical proteins account for around 20-40% of the total genome [42, 71], which are important for complementing the genomic and proteomic framework theory. Understanding the functional properties of these proteins will be crucial for a more profound understanding of the microbes at the molecular machinery level. For pathogens, those proteins with unknown functions probably involve in virulence which help elucidating pathogenesis and are virgin area for developing novel drug/vaccine targets.

In our study, 45 hypothetical proteins and 78 uncharacterized proteins were identified. Among them, 11 uncharacterized/hypothetical proteins displayed differential expression levels, 8 were overrepresented, and 3 were underrepresented in strain 168. It is reasonable for us to suppose that the differentially expressed proteins of unknown function might give a clue for pathogenic mechanisms of* M. hyopneumoniae* or novel drug/vaccine targets. The VirulentPred results showed that all the overrepresented proteins in strain 168 among the differentially expressed uncharacterized/hypothetical proteins are predicted to be associated with virulence. Thus the protein sequences were submitted to BlastP, Pfam, and InterPro web servers for putative function annotation.  Table 5 shows the results of the BlastP, Pfam, and InterPro. We also performed the prediction of subcellular localization as a complement to facilitate our knowledge of these uncharacterized/hypothetical proteins (Table S4). However, experimental studies should be carried out to assess and confirm their functions in biological processes and pathogenesis.

Proteins ADQ90691, ADQ90727, and ADQ90524 were annotated as hydrolase enzymes. Members of this class are involved in various significant biological processes, including virulence mechanisms [42, 72]. ADQ90691 was significantly expressed in virulent strain and predicted as a virulence factor, probably playing an important role in the pathogenesis of* M. hyopneumoniae*. This protein was annotated to be a Cof-like hydrolase, while might function as the phosphatase Cof in* E.coli*, catalyzing the hydrolysis of 4-amino-2-methyl-5-hydroxymethylpyrimidine pyrophosphate to 4-amino-2-methyl-5-hydroxymethylpyrimidine phosphate [73].

The hypothetical protein ADQ90530 was categorized as a sirtuin (also known as Sir2), which is responsible for NAD^+^-dependent deacetylation. As the deacetylase of* M. fermentans* is expressed inside mammalian cells, it inhibits cell proliferation but promotes their antioxidation and antistarvation capacities, and alters gene expression, affecting physiological functions and the corresponding signal transduction pathways in host cells [74]. The long-held view is that* M. hyopneumoniae* is an extracellular pathogen, thus it remains unclear whether ADQ90530 in* M. hyopneumoniae* functions similarly with secreted deacetylase in intracellular pathogen* M. fermentans*. Elucidating this issue will require further investigation.

Protein ADQ90824 was predicted to be an N-6 adenine-specific DNA methylase, which controls methylation at adenine residues of important biological processes. The methylation process plays an important role in bacterial pathogenesis by regulating the synthesis of virulence factors, and DNA adenine methylases serve as promising antimicrobials and vaccines targets [42, 75].

Leucine-rich repeats (LRRs) are found to be present in a number of proteins with diverse functions, including cell-adhesion molecules, virulence factors, and extracellular matrix-binding glycoproteins, and function in signal transduction, cell-adhesion, and protein-protein interactions [76, 77]. Protein ADQ90745 containing a leucine-rich repeat domain is predicted to be related to virulence, yet more detailed information about its function is unavailable so far.

Uncharacterized protein ADQ90859 belongs to the mycoides cluster lipoprotein, LppA/p72 family; members of this protein family are predicted lipoproteins with a typical prokaryotic signal peptidase II processing and lipid attachment site [78]. Paralogues in other mycoplasmas have been identified as specific antigenic proteins with potential for use in development of diagnostic reagents [78, 79].

Hypothetical protein ADQ90910 was classified as one member (H-protein) of the glycine cleavage system composed of four proteins: the T-, P-, L-, and H-protein. This system catalyzes the reversible reaction: Glycine + H_4_folate + NAD^+^ <==> 5, 10-methylene-H_4_folate + CO_2_ + NH_3_ + NADH + H^+^, and H-protein shuttles some of the intermediate products [80]. H-protein was expressed exclusively in* Francisella tularensis* isolated from mouse spleens compared with* in vitro* grown controls, suggesting that H-protein may play an important role in the metabolic fitness of* Francisella tularensis* [81]. A similar result of predicted H-protein was detected in proteomic analysis of* M. hyopneumoniae*; we assumed hypothetical protein ADQ90910 also play a part in the adaptive response of pathogen to* in vivo* environment.

The “DUF31” domain that has no known function was found in uncharacterized protein ADQ90827, and none of the domains was predicted in hypothetical protein ADQ90324. The function of them cannot be identified but is predicted to be associated with virulence.

## 5. Conclusions

In conclusion, this survey identified mycoplasmal proteins and unveiled biological processes potentially related to the difference in virulence between the pathogenic and attenuated strains of* M. hyopneumoniae*. The components that play roles in these critical mechanisms are natural targets for specific drugs. These potential virulence factors are usually immunogenic and can be treated as vaccine candidates [82, 83]. Our future experimental work will focus on those proteins to delineate the pathogenic mechanism(s) and identify those which can be targeted for drug design and vaccine development.

## Figures and Tables

**Table 1:** Primers used for amplication and site-directed mutagenesis.

Target	Primers	Sequence (5′ to 3′)	Description
Endo-1,4-beta-glucanase	MHP168_174-F	CGC*GGATCC*ATGTCAATATTAGAAAAAAT	Amplification
	MHP168_174-M1R	CGCATTTCCcCAAATTCCGC	Site directed mutagenesis
	MHP168_174-M1F	GCGGAATTTGgGGAAATGCG	Site directed mutagenesis
	MHP168_174-R	CGC*CTCGAG*TTATTTATATATTAACTC	Amplification

Enolase	MHP168_271-F	CTT*GGATCC*ATGTCAAAAATTACTAAAG	Amplification
	MHP168_271-M1R	CTTTTTGACCATAcCAATTATCTGC	Site directed mutagenesis
	MHP168_271-M1F	GCAGATAATTGgTATGGTCAAAAAG	Site directed mutagenesis
	MHP168_271-M2R	GATAAAACCCTGcCAATCAGATTC	Site directed mutagenesis
	MHP168_271-M2F	GAATCTGATTGgCAGGGTTTTATC	Site directed mutagenesis
	MHP168_271-R	TAT*CTCGAG*CTATTTTTTTAAGTTATAAAAAACT	Amplification

Translation elongation factor Tu	MHP168_533-F	CTC*GGATCC*ATGGCAGTTGTTAAAACG	Amplification
	MHP168_533-R	CCG*CTCGAGT*TATTTAATAATTTCGGT	Amplification

Methylmalonate-semialdehyde dehydrogenase	MHP168_244-F	CGGGGATCCATGAATTTTAAAAGAAATTAC	Amplification
	MHP168_244-M1R	CATTTGGAATTGAcCAATTTGGCAC	Site directed mutagenesis
	MHP168_244-M1F	GTGCCAAATTGgTCAATTCCAAATG	Site directed mutagenesis
	MHP168_244-M2R	CGGCTTGTTTcCAAAGATCAG	Site directed mutagenesis
	MHP168_244-M2F	CTGATCTTTGgAAACAAGCCG	Site directed mutagenesis
	MHP168_244-M3R	GAATCTCTGAcCAGTCAATAATTG	Site directed mutagenesis
	MHP168_244-M3F	CAATTATTGACTGgTCAGAGATTC	Site directed mutagenesis
	MHP168_244-M4R	AATCAAATTAGTcCAGAAATTAATACTGG	Site directed mutagenesis
	MHP168_244-M4F	CCAGTATTAATTTCTGgACTAATTTGATT	Site directed mutagenesis
	MHP168_244-M5R	CTTCCGGGTTcCATTTTGTAG	Site directed mutagenesis
	MHP168_244-M5F	CTACAAAATGgAACCCGGAAG	Site directed mutagenesis
	MHP168_244-R	CTTCTCGAGTTATGACATcCAATCCTG	Amplification and site directed mutagenesis

**Table 2:** Proteins differentially expressed between the compared samples *(M. hyopneumonia*e strain 168 and strain 168L).

Identified Proteins	Accession Number	Gene_locus	Gene	log⁡2 ratios (168/168L)	Unique Peptide	Percentage Coverage
*proteins which were more abundant in 168L*						

Myo-inositol catabolism protein	ADQ90457	MHP168_246	-	-3.04	10	45%

Myo-inositol 2-dehydrogenase	ADQ90458	MHP168_247	-	-2.89	16	43%

Periplasmic sugar-binding protein	ADQ90463	MHP168_252	rbsB	-2.76	21	61%

Methylmalonate-semialdehyde dehydrogenase	ADQ90455	MHP168_244	-	-2.53	21	48%

Serine hydroxymethyltransferase	ADQ90454	MHP168_243	glyA	-2.4	14	36%

5-Dehydro-2-deoxygluconokinase	ADQ90456	MHP168_245	iolC	-2.25	11	30%

Myo-inositol 2-dehydrogenase	ADQ90464	MHP168_253	-	-2.07	11	39%

Myo-inositol catabolism	ADQ90459	MHP168_248	iolD	-2.04	17	27%

Myo-inositol catabolism protein	ADQ90460	MHP168_249	iolE	-2.04	5	20%

Site-specific DNA-methyltransferase	ADQ90525	MHP168_318	mod	-1.65	7	27%

Hypothetical protein	ADQ90289	MHP168_066	-	-1.54	5	5.80%

Ribose ABC transport ATP-binding protein	ADQ90461	MHP168_250	mglA	-1.39	14	35%

Lipoprotein	ADQ90594	MHP168_392	-	-1.21	36	54%

Putative uncharacterized protein	ADQ90727	MHP168_531	-	-1.21	63	33%

Protein P97-copy 2	ADQ90409	MHP168_195	-	-1.12	34	38%

Glucose kinase	ADQ90722	MHP168_526	glcK	-1.04	7	25%

Protein p97, cilium adhesin	ADQ90328	MHP168_110	-	-1.03	70	64%

tRNA uridine 5-carboxymethylaminomethyl modification enzyme mnmG	ADQ90231	MHP168_003	gidA	-1.01	12	20%

Neutrophil activating factor	ADQ90913	MHP168_736	-	-0.98	6	41%

Xylose ABC transporter ATP-binding protein	ADQ90719	MHP168_523	xylG	-0.93	15	35%

Xylose ABC transporter permease protein	ADQ90720	MHP168_524	xylH	-0.93	4	8.20%

ABC transporter ATP-binding protein	ADQ90246	MHP168_019	-	-0.91	6	8.50%

Lipoprotein	ADQ90595	MHP168_393	-	-0.88	9	28%

GTPase obg	ADQ90265	MHP168_042	obgE	-0.88	6	17%

Protein P102-copy 2	ADQ90410	MHP168_196	-	-0.83	21	25%

50S ribosomal protein L24	ADQ90339	MHP168_121	rplX	-0.81	6	48%

Heat-inducible transcription repressor hrcA	ADQ90238	MHP168_011	hrcA	-0.78	13	36%

50S ribosomal protein L23	ADQ90331	MHP168_113	rplW	-0.77	11	56%

Thioredoxin	ADQ90613	MHP168_411	-	-0.77	11	91%

Putative uncharacterized protein	ADQ90524	MHP168_317	-	-0.76	13	16%

DNA topoisomerase I	ADQ90512	MHP168_305	topA	-0.71	16	28%

50S ribosomal protein L14	ADQ90338	MHP168_120	rplN	-0.71	10	64%

Holliday junction ATP-dependent DNA helicase ruvA	ADQ90632	MHP168_430	ruvA	-0.67	8	36%

Transcription termination-antitermination factor nusA	ADQ90786	MHP168_593	nusA	-0.65	21	34%

50S ribosomal protein L3	ADQ90329	MHP168_111	rplC	-0.6	14	58%

*proteins which were more abundant in 168*						

Triacylglycerol lipase	ADQ90483	MHP168_274	lip	0.59	13	46%

Putative uncharacterized protein	ADQ90691	MHP168_495	-	0.59	8	30%

Enolase	ADQ90480	MHP168_271	eno	0.61	14	48%

Translation elongation factor Tu	ADQ90729	MHP168_533	tuf	0.61	23	66%

Hypoxanthine phosphoribosyltransferase	ADQ90492	MHP168_283	hpt	0.61	9	50%

Transport protein sgaT	ADQ90622	MHP168_420	ulaA	0.62	7	13%

Cation-transporting P-type ATPase	ADQ90498	MHP168_289	pacL	0.63	8	13%

Hypothetical protein	ADQ90530	MHP168_323	-	0.63	5	14%

Putative uncharacterized protein	ADQ90824	MHP168_634	-	0.66	22	28%

Putative uncharacterized protein	ADQ90745	MHP168_549	-	0.68	11	46%

Acetate kinase AckA	ADQ90711	MHP168_515	ackA	0.69	17	54%

Cysteinyl-tRNA synthetase	ADQ90838	MHP168_650	cysS	0.69	7	15%

Putative uncharacterized protein	ADQ90859	MHP168_673	-	0.7	11	28%

Pts system, lichenan-specific IIa component	ADQ90264	MHP168_041	licA	0.71	9	33%

Probable O-sialoglycoprotein endopeptidase	ADQ90833	MHP168_644	gcp	0.73	10	37%

CTP synthase	ADQ90509	MHP168_302	pyrG	0.74	14	25%

Lipoate-protein ligase A	ADQ90531	MHP168_324	lplA-1	0.75	14	33%

ABC transporter ATP-binding-Pr1	ADQ90875	MHP168_691	pr1	0.75	7	16%

Hypothetical protein	ADQ90910	MHP168_731	-	0.76	4	35%

Thymidine kinase	ADQ90808	MHP168_618	tdk	0.78	8	40%

Lipoate-protein ligase A	ADQ90484	MHP168_275	lplA	0.78	5	16%

Purine-nucleoside phosphorylase	ADQ90309	MHP168_087	deoD	0.8	7	29%

Endo-1,4-beta-glucanase	ADQ90389	MHP168_174	-	0.84	20	64%

NADH dependent flavin oxidoreductase	ADQ90532	MHP168_325	baiH	0.85	4	11%

ABC transporter protein	ADQ90806	MHP168_616	-	0.87	6	11%

Sugar ABC transporter ATP-binding protein	ADQ90805	MHP168_615	-	0.9	10	26%

MG2+ ion transporter	ADQ90693	MHP168_497	mgtE	0.9	9	23%

Ribonuclease III	ADQ90602	MHP168_400	rnc	0.94	9	34%

Ribose-phosphate pyrophosphokinase	ADQ90852	MHP168_664	prsA	0.98	12	39%

Glycerol transporter subunit A	ADQ90619	MHP168_417	gtsA	1.13	4	11%

Putative uncharacterized protein	ADQ90827	MHP168_638	-	1.16	4	7.50%

hypothetical protein	ADQ90324	MHP168_104	-	1.59	19	23%

Glycerol kinase	ADQ90587	MHP168_385	glpK	1.59	22	49%

Lipoprotein	ADQ90620	MHP168_418	-	1.7	31	55%

PTS system galactitol-specific enzyme IIB component	ADQ90760	MHP168_564	-	1.78	4	57%

**Table 3:** Bioinformatic analyses for the proteins selected for validation.

Protein	Number of amino acids	Molecular weight	Theoretical PI	Number of predicted TMHs	Signal peptide	Number of TGA codons
Endo-1,4-beta-glucanase	356 aa	39.2kDa	6.21	0	No	1

Enolase	452 aa	49.5kDa	5.83	0	No	2

Translation elongation factor Tu	402 aa	44.1kDa	5.61	0	No	0

Methylmalonate-semialdehyde dehydrogenase	489 aa	53.9kDa	8.46	0	No	6

**Table 4:** VirulentPred screening of the 35 overrepresented proteins in *M. hyopneumoniae* 168. References supporting the association with virulence are included.

Identified Proteins	Accession Number	Prediction results	Reference
Putative uncharacterized protein	ADQ90691	Virulent	Okan et al. 2013; Shahbaaz et al. 2015

Hypothetical protein	ADQ90530	Virulent	Cheng et al. 2017

Putative uncharacterized protein	ADQ90824	Virulent	Heithoff et al. 1999; Shahbaaz et al. 2015

Putative uncharacterized protein	ADQ90745	Virulent	Gay et al. 1991; Kobe et al. 2001

Cysteinyl-tRNA synthetase	ADQ90838	Virulent	

Putative uncharacterized protein	ADQ90859	Virulent	Cheng et al. 1996; Monnerat et al. 1999

Pts system, lichenan-specific IIa component	ADQ90264	Virulent	

ABC transporter ATP-binding-Pr1	ADQ90875	Virulent	Garmory et al. 2004

Hypothetical protein	ADQ90910	Virulent	Twine et al. 2006

ABC transporter protein	ADQ90806	Virulent	Garmory et al. 2004

Sugar ABC transporter ATP-binding protein	ADQ90805	Virulent	Garmory et al. 2004

MG2+ ion transporter	ADQ90693	Virulent	Groisman et al. 2013; Merino et al. 2001; O'Connor et al. 2009

Ribonuclease III	ADQ90602	Virulent	Haddad et al. 2013; Mao et al. 2016

Ribose-phosphate pyrophosphokinase	ADQ90852	Virulent	Donini et al. 2017

Putative uncharacterized protein	ADQ90827	Virulent	

Hypothetical protein	ADQ90324	Virulent	

Lipoprotein	ADQ90620	Virulent	

PTS system galactitol-specific enzyme IIB component	ADQ90760	Virulent	

Enolase	ADQ90480	Nonvirulent	Bao et al. 2014; Pancholi 2001; Schreiner et al. 2012

Translation elongation factor Tu	ADQ90729	Nonvirulent	Dallo et al. 2002; Jiang et al. 2016

Cation-transporting P-type ATPase	ADQ90498	Nonvirulent	Novoa-Aponte et al. 2014

Probable O-sialoglycoprotein endopeptidase	ADQ90833	Nonvirulent	Aruni et al. 2011; Davis et al. 2003

Lipoate-protein ligase A	ADQ90531	Nonvirulent	O'Riordan et al. 2003

Lipoate-protein ligase A	ADQ90484	Nonvirulent	O'Riordan et al. 2003

Endo-1,4-beta-glucanase	ADQ90389	Nonvirulent	Robinson et al. 2013

Acetate kinase AckA	ADQ90711	Nonvirulent	

NADH dependent flavin oxidoreductase	ADQ90532	Nonvirulent	

Glycerol transporter subunit A	ADQ90619	Nonvirulent	

Glycerol kinase	ADQ90587	Nonvirulent	

Triacylglycerol lipase	ADQ90483	Nonvirulent	

Hypoxanthine phosphoribosyltransferase	ADQ90492	Nonvirulent	

Transport protein sgaT	ADQ90622	Nonvirulent	

CTP synthase	ADQ90509	Nonvirulent	

Thymidine kinase	ADQ90808	Nonvirulent	

Purine-nucleoside phosphorylase	ADQ90309	Nonvirulent	

**Table 5:** List of predicted functions of differentially expressed uncharacterized/hypothetical proteins.

Accession Number	BlastP	pfam	InterPro
*proteins which were more abundant in 168*			

ADQ90691	Cof-type HAD-IIB family hydrolase	Hydrolase_3 Domain	None predicted.

ADQ90530	deacetylase SIR2	No result	Sirtuin family, catalytic core domain

ADQ90824	SAM-dependent DNA methyltransferase	N6_Mtase Family	DNA methylase, adenine-specific

ADQ90745	Leucine-rich repeat domain-containing protein	LRR_5 Repeat	BspA type Leucine rich repeat region

ADQ90859	LppA family lipoprotein	No result	None predicted

ADQ90910	Glycine cleavage system protein H	GCV_H Domain	None predicted

ADQ90827	DUF31 domain-containing protein	DUF31 Domain (Putative peptidase)	Putative peptidase DUF31

ADQ90324	No result	No result	None predicted

*proteins which were more abundant in 168L*			

ADQ90289	SGNH/GDSL hydrolase family protein	No result	Bromodomain

ADQ90727	SGNH/GDSL hydrolase family protein	Lipase_GDSL Family	GDSL lipase/esterase

ADQ90524	DEAD/DEAH box helicase	ResIII Family	Helicase/UvrB, N-terminal

## Data Availability

The data used to support the findings of this study are included within the article.

## Supplementary Materials

Supplementary 1 Table S1: information of all proteins identified in this study.

Supplementary 2 Table S2: Gene Ontology enrichment analysis for differentially expressed proteins.

Supplementary 3 Table S3: pathway enrichment analysis of the annotated differentially expressed proteins.

Supplementary 4 Table S4: bioinformatic analyses of the differentially expressed uncharacterized/hypothetical proteins.
